# An analysis of the significance of the Tre2/Bub2/CDC 16 (TBC) domain protein family 8 in colorectal cancer

**DOI:** 10.1038/s41598-022-15629-1

**Published:** 2022-08-02

**Authors:** Yuan-jie Liu, Jie-pin Li, Hui-ru Li, Shu-hong Zeng, Yu-gen Chen, Mei Han, Qian-wen Ye, Jin-yong Zhou, Shen-lin Liu, Xi Zou

**Affiliations:** 1 Affiliated Hospital of Nanjing University of Chinese Medicine, Jiangsu Province Hospital of Chinese Medicine, Nanjing, 210029 Jiangsu China; 2 Department of Oncology, Zhangjiagang TCM Hospital Affiliated to Nanjing University of Chinese Medicine, Zhangjiagang, 215600 Jiangsu China; 3 No. 1 Clinical Medical College, Nanjing University of Chinese Medicine, Nanjing, 210023 Jiangsu China

**Keywords:** Cancer, Cell biology, Genetics, Oncology

## Abstract

The TBC (Tre-2/Bub2/Cdc16, TBC) structural domain is now considered as one of the factors potentially regulating tumor progression. However, to date, studies on the relationship between TBC structural domains and tumors are limited. In this study, we identified the role of TBC1 domain family member 8 (*TBC1D8*) as an oncogene in colorectal cancer (CRC) by least absolute shrinkage and selection operator (LASSO) and Cox regression analysis, showing that *TBC1D8* may independently predict CRC outcome. Functional enrichment and single-cell analysis showed that *TBC1D8* levels were associated with hypoxia. *TBC1D8* levels were also positively correlated with M2 macrophage infiltration, which may have a complex association with hypoxia. Taken together, these results show that the *TBC1D8* gene is involved in colorectal carcinogenesis, and the underlying molecular mechanisms may include hypoxia and immune cell infiltration.

## Introduction

Colorectal cancer (CRC) is a tumor of the gastrointestinal system, ranking third in incidence among malignancies and second in lethality among cancers^1^. Its pathogenesis appears to involve multiple factors and steps^2^, including genetics, diet, and lifestyle habits^3,4^. Statistics show that the tumor frequently recurs and metastasizes, spreading especially to the liver, lung, bone, ovaries, peritoneal and the five-year survival rate for such patients is approximately 10%^5,6^. Treatment methods for CRC include chemotherapy, surgery, radiotherapy, and molecular-targeted therapy^7,8^. Molecular-targeted therapy for CRC has become a hot topic^9,10^. The number of molecularly stratified treatment options is increasing, as is the use of biomarkers to guide prediction and treatment decisions^11^. Therefore, the identification of novel markers and targets for treating CRC is necessary for improving the disease prognosis.

The TBC (Tre-2/Bub2/Cdc16, TBC) structural domain is a conserved amino acid sequence consisting of the oncogene Tre-2 (gene symbol: *USP6*) and the structural domains of the yeast cell circulation regulator genes Bub2 and Cdc16^12^. TBC-domain family members are strongly conserved with high levels of homology^13^. TBC structural domains are often located in tandem with other structural domains associated with cell membrane functions, suggesting that the biological functions of TBC structural domain proteins are closely linked to cell membranes and may mediate the onset and progression of cancer or other diseases^14–16^ and, indeed, an association with tumor progression has been found for a number of these proteins. High *TBC1D7* levels have been observed in several lung cancers and have been correlated with poor patient outcomes. RNAi interference of *TBC1D7* in lung cancer cells inhibited cell growth; conversely, overexpression of *TBC1D7* promoted lung cancer cell proliferation and promoted tumor formation in mice^17^. *TBC1D16* also appears to be the driving gene in melanoma, and its overexpression can promote cell growth and gene expression, affecting vesicle transport and further influencing melanoma progression^18^. It has been shown that *TBC1D8* is significantly up-regulated in invasive ovarian cancer cells, which drives the occurrence and metabolic reprogramming of ovarian cancer^19^. However, there are few studies on its role in CRC. The present study seeks to fill this lacuna.

In this study, human CRC specimens were analyzed by immunohistochemistry. At the same time, using bioinformatics software and methods, we performed an initial exploration of the potential function and mechanisms of *TBC1D8*.

## Materials and methods

### Reagents

The reagents and antibodies used are listed in the Supplementary material (Supplementary Table 1). The concentrations used were based on those used in previous research or on the manufacturers’ recommendations. Experimental procedures are also provided in the Supplementary material.

### Cell culture

The human CRC cell lines, SW620, SW480, RKO, HCT116, HT-29, and LoVo, as well as the human normal human colonic epithelial cell line, NCM460, and THP-1 cells (human monocytic cells) were acquired from the cell bank of the Chinese Academy of Sciences (Shanghai, China). SW620 cells were grown in Leibovitz's L-15 medium with 10% fetal bovine serum (FBS), while the other lines were grown in DMEM with 10% FBS at 5% CO_2_ and 37 °C. All media were supplemented with 1% penicillin/streptomycin. Cells passed regular mycoplasma contamination testing.

### Ethics statement and specimen collection

The study’s protocol was approved by the ethics committee of the Jiangsu Province Hospital of Chinese Medicine, and informed consent was obtained from clinicians and patients (2020NL-107-01). All experiments were conducted in accordance with the Declaration of Helsinki and in accordance with the relevant designated guidelines and regulations. Written information was provided and written consent was given to all participants and informed consent was obtained from all participants prior to collection of specimens^20^. Thirty CRC tissues and paracancerous tissues (5-cm margin) were obtained intra-operatively from untreated patients at the Jiangsu Province Hospital of Chinese Medicine. Tumors were scored using the 8th edition of the “American Joint Committee on Cancer tumor-node-metastasis” (TNM) system^21^. Samples were washed in chilled PBS and quick-frozen in liquid nitrogen after which they were stored at − 80 °C until analysis.

### Immunohistochemical (IHC) staining

IHC was conducted according to a previously published protocol^22^. Sections were blocked with blocking solution and incubated with anti-TBC1D8 antibodies. The extent and intensity of staining were assessed by two independent investigators. Staining intensities were graded as 0 (none), 1 (weak), 2 (moderate), and 3 (strong). Staining extent was graded as 0 (no positively-stained cells), 1 (less than 10%), 2 (10–50%), and 3 (over 50%). The histochemistry score (H-SCORE), representing both the proportion of stained cells and the degree of staining, was determined as: “H-SCORE = ∑ (PI × I) = (percentage of cells with weak intensity × 1) + (percentage of cells with moderate intensity × 2) + (percentage of cells with strong intensity × 3)”, where PI represents the percentage of positive cells to the total number of cells in a particular field and I represent the intensity of staining. The H-SCORE ranged between 0 and 300, with higher scores indicating more intense staining.

### Establishment of hypoxia model

A stock solution of CoCl_2_ was prepared by dissolving solid CoCl_2_ in serum-free DMEM. Using a previously published method^23^, we pretreated cells with 300 µmol/L CoCl_2_ for 30 h. CoCl_2_ induces hypoxia through blocking degradation of HIF-α, activating the hypoxia cascade^24^.

### TBC1D8 expression assessment

The Western blotting method followed previous descriptions^25^. Proteins were lysed in RIPA buffer and protein concentrations were measured by the Bradford assay. Samples of 20 µg each were separated on 10% or 8% SDS-PAGE, transferred to polyvinylidene fluoride (PVDF) membranes, and blocked with 5% bovine serum albumin. The blots were then probed with the relevant primary antibodies at 4 °C overnight. Blots were washed in Tris-buffered saline containing 0.05% Tween-20 and were incubated with the corresponding secondary antibodies with an Electrochemiluminescence (ECL) detection kit used to measure densities. The protein bands (including β-actin as the loading control) were visualized with a gel image processing system (ChemiDoc XRS +) and relative concentrations were determined. The expression levels of each protein were normalized relative to β-actin protein expression and are shown as relative protein levels. Relative protein expression = gray value of target protein band/gray value of β-actin band. TBC1D8 expression in CRC was first investigated using the Tumor Immune Estimation Resource (TIMER) web tool (https://cistrome.shinyapps.io/timer/) Cancer Cell Line Encyclopedia (CCLE) database (https://portals.broadinstitute.org/ccle); The Human Protein Atlas (HPA) (https://www.proteinatlas.org/) and the Gene Expression Profiling Interactive Analysis (GEPIA) database (https://www.oncomine.org/).

### RNAi plasmids construction and transfection

HCT116 and HT-29 cells which express relatively high levels of TBC1D8 were selected for subsequent experiments. The plasmids described below were all constructed by GeneChem (Shanghai, China). The procedures for constructing RNAi plasmids are described in the Supplementary material. From three short hairpin interfering RNA (shRNA) targeting TBC1D8, we selected the one with the highest inhibitory efficiency for the subsequent experiments. The shRNA-TBC1D8 plasmid (sh-TBC1D8) and the control (NC) non-targeting sequence plasmid were transfected into 70%-confluent CRC cells using Lipofectamine 3000 per provided protocols. The transduction efficiencies were examined by western blotting and Green fluorescent protein (GFP) expression.

### Colony formation assays

The ability of cells to form colonies was measured as previously described^26^. Five hundred cells per well were plated in 6-well plates and grown for approximately 14 days. Colonies (a minimum of five cells) were stained with 0.5% crystal violet at room temperature (20–25 °C) for 10 min and counted under light microscopy (Olympus BX53, Japan).

### Xenograft tumor model

All animal experiments were performed according to the “Guide for the Care and Use of Laboratory Animals” (NIH Publications No. 80‐23, revised 1996) and approved by the ethics committee of the Jiangsu Province Hospital of Chinese Medicine (2021-11-032). 4-week-old male BALB/c nude mice were acquired from the Beijing Institute of Biomedicine (Beijing, China) (Certificate No. SYXK2019-0010). Cells (HCT-116 cells transfected with sh-TBC1D8 and NC cells; 1 × 10^7^ cells/mouse) were injected subcutaneously into the right armpit region (n = 6 per group). Tumors were visible after seven days. Tumor diameters (maximum and minimum) were measured twice a week. The mice were euthanized under CO_2 _on day 35, and the sera and tumors were excised. Euthanasia was fully consistent with the recommendations of the Guidelines on Euthanasia of the American Veterinary Medical Association. Tumor volumes were calculated as V = 1/2ab^2^, and tumor growth curves were drawn.

### Sphere formation

Sphere formation was examined as previously described^27^. Single-cell suspensions were plated in 6-well ultralow attachment plates (5 × 10^3^ cells/well) and grown in serum-free medium with Human recombinant Fibroblast growth factor (FGF) (20 ng/ml), Human recombinant Epidermal Growth Factor (EGF) (20 ng/ml), and 2% B27. The numbers and sizes of the spheres were determined after seven days under light microscopy.

### Co-culture system

Cell slides were placed at the bottom of the 6-well plates at the beginning of the co-culture experiment. THP-1 monocytic cells (1 × 10^5^ cells/mL) were incubated with 10 ng/mL PMA for 48 h to allow differentiation into M0 macrophages^27^ The medium was then replaced with serum-free DMEM and the cells were allowed to grow for 24 h. Co-cultures between M0 and CRC were conducted using Transwell inserts (0.4 µm) in which culture medium was diffusible but cells were not permeable. To mimic the interactions between macrophages and tumors, 6 × 10^5^ CRC cells were placed in the upper chamber of a 6-well transwell apparatus and the M0 macrophages were placed in the lower chamber.

### Immunofluorescence

Immunofluorescence staining was conducted according to a previously published protocol^28^. Briefly, the cells cultured on cover slips were fixed, permeabilized with 0.5% Triton X-100, and incubated with anti-CD163 and anti-CD206 polyclonal antibodies overnight at 4 °C. Subsequently, the slides were incubated with secondary antibodies. Nuclei were stained using 4',6-diamidino-2-phenylindole (DAPI) for 3 min, and the cells were incubated in the dark for 3 min. The slides were washed with PBS four times, for 5 min each. Then, the slides were sealed with sealing solution containing a fluorescence quencher and observed and imaged under a fluorescence microscope. Immunofluorescence was examined under an epi-fluorescence microscope (Olympus CKX-41,). By changing the filters, the different secondary antibodies could be identified in double-stained sections. Images were captured with a digital camera (Olympus, DP50).

### Transcriptomic expression and survival analysis

*TBC1D8* expression was investigated using the The Cancer Genome Atlas (TCGA)-Colon Adenocarcinoma (COAD) cohort and the GSE10950 and GSE37182 datasets^29^. Expression levels were analyzed in terms of different parameters, namely, “T (Tumor)”, “N (Node)”, and “M (Metastasis)” stages using the TCGA-COAD data.

Survival analysis, specifically, the Overall Survival (OS), disease-specific survival (DSS), and progression-free interval (PFI) were performed by log-rank test using the TCGA-COAD data.

### LASSO and cox regression

Raw RNA-seq and clinical data were acquired from the TCGA dataset in January 2020. LASSO regression analysis was conducted using the R package ‘glmnet' to determine the research objective. Univariate and multivariate Cox regression was performed to determine the independent prognostic factors of *TBC1D8*. The forest plot representing the P-value, hazard ratios (HRs), and 95% confidence intervals (CIs) of each variable were determined using the “forestplot” package in R.

### Function analysis of TBC1D8-associated genes

The median cutoff of *TBC1D8* expression in the TCGA-COAD was used to differentiate high and low *TBC1D8* groups. The R “limma” package was used to identify *TBC1D8*-associated genes^30^. Enrichment analysis was conducted with the “clusterProfiler” package in R to examine possible *TBC1D8* functions^31^.

Gene-Set Enrichment Analysis (GSEA) was conducted with the Broad Institute GSEA software 3.0^32^. The gene set “Gene Ontology (GO)” was downloaded from the Molecular Signatures Databases (http://www.gsea-msigdb.org/gsea/msigdb/index.jsp) and was used for GO enrichment. A False Discovery Rate (FDR) < 0.1 represented statistical significance.

For Gene Set Variation Analysis (GSVA)^33^, the R package “GSVA” was used to conduct enrichment analysis and “Limma” was used to identify differentially enriched genesets based on TCGA-COAD and GSE37182. To further explore the association between TBC1D8 and hypoxia, we defined 29 prognosis-related hypoxia genes with reference to the work of Joon-Hyop Lee et al.^34^. and calculated the correlation between them and TBC1D8.The Tumor Immune Single Cell Hub (TISCH)^35^ based GSE146771 data and the CancerSEA^36^ were used for the single-cell level comprehensive exploration for cancer cell function^37^.

### Immune analysis

The association between *TBC1D8* level and immune infiltration was examined with the Estimating the Proportion of Immune and Cancer cells (EPIC) algorithm based on TCGA-COAD^38^. TIMER (TCGA-COAD) was used for reliable assessment of infiltration, pairwise comparisons were quantified by Spearman’s rank correlation, with P < 0.05 representing significance. CIBERSORT was used to confirm the relationship between *TBC1D8* levels and macrophage polarization^39^ based on. GSE10950 and GSE37182 All results were processed using the “ggplot2” and “pheatmap” packages in R.

### DNA methylation and genetic alternation analysis

The analysis of *TBC1D8* DNA methylation and the correlation between disease prognosis and *TBC1D8* methylation values was performed using the Methsurv and MEXPRESS databases (http://mexpress.be). We also used the SurvivalMeth database^40^ to analyze the differential methylation levels of different probes for *TBC1D8* between normal and tumor tissues. The alteration frequency of the *TBC1D8* gene in several studies of CRC, including the Colon Cancer (CPTAC-2 Prospective, Cell 2019), Colorectal Adenocarcinoma (Genentech, Nature 2012), Colorectal Adenocarcinoma (DFCI, Cell Reports 2016), Colorectal Adenocarcinoma (TCGA, PanCancer Atlas), Colorectal Adenocarcinoma (TCGA, Nature 2012), Colorectal Adenocarcinoma (TCGA, Firehose Legacy), Colon Adenocarcinoma (CaseCCC, PNAS 2015), and Metastatic Colorectal Cancer (MSKCC, Cancer Cell 2018)) studies, was analyzed via the cBioPortal database (http://www.cbioportal.org). We provided data of genomic alteration type, mutation site profile analyses. In addition, we analyzed the differential expression of macrophage markers in the wild-type and mutant *TBC1D8* groups using the TIMER database (https://cistrome.shinyapps.io/timer/).

### Statistical analysis

Data were expressed as means ± SEM. Comparisons between two groups and multiple groups were assessed by t-tests and one-way ANOVA, respectively. All data were analyzed with SPSS 29.0 (IBM Corp., Armonk, NY, USA) and visualized with GraphPad Prism 8.0 (GraphPad Software, Inc., USA). Experiments were conducted a minimum of three times. ****P < 0.0001, ***P < 0.001, **P < 0.01 and *P < 0.05 were defined to be statistically significant.

### Adherence to ARRIVE guidelines

Our study complies with the ARRIVE guidelines (https://arriveguidelines.org).

## Results

### Prognostic value of TBC domain family members

**Figure 1:**
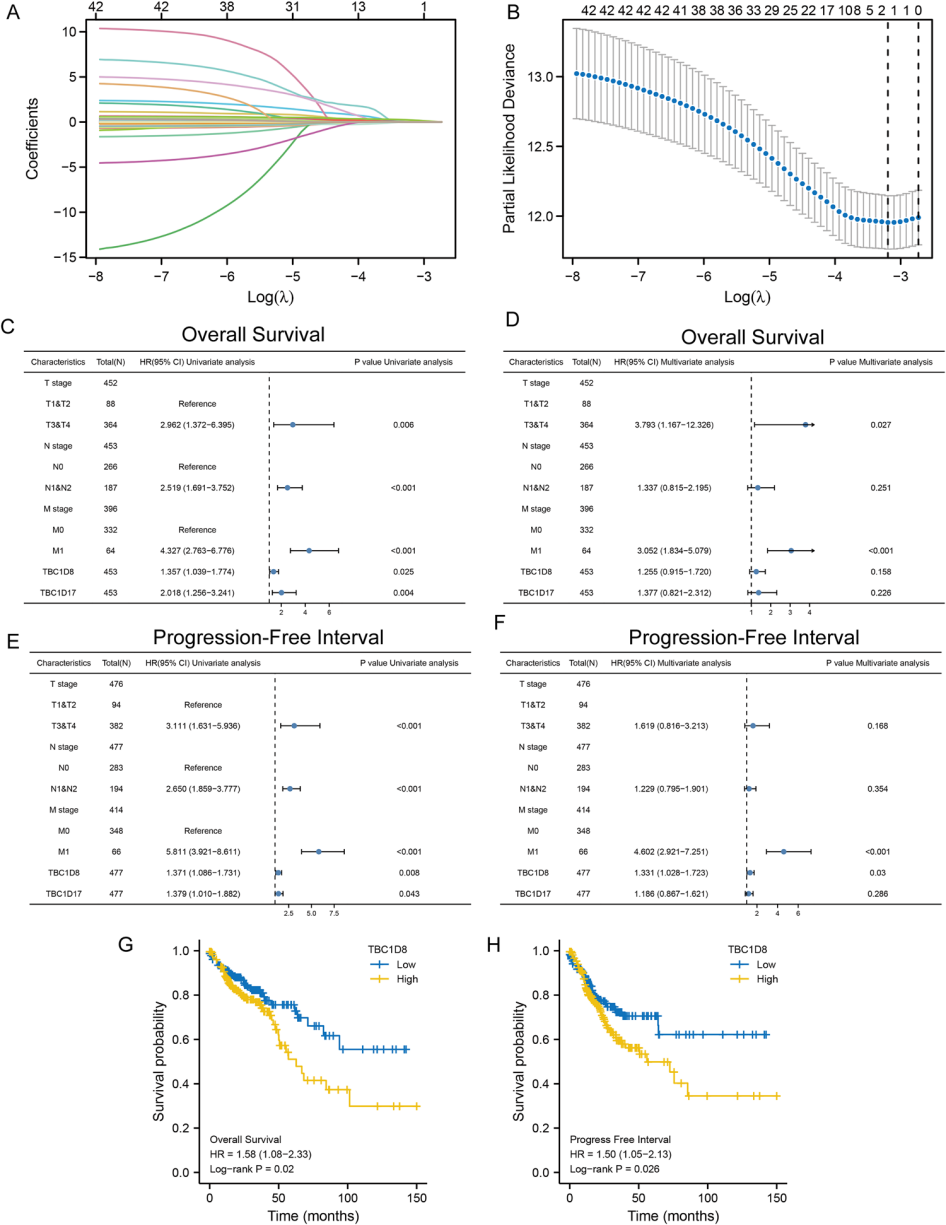
*TBC1D8* expression as an indicator of overall survival and progression-free interval determined by Least absolute shrinkage and selection operator (LASSO) and Cox regression analysis in the The Cancer Genome Atlas (TCGA) cohort. (**A**) In the LASSO-Cox model based on TCGA-Colon Adenocarcinoma (COAD), the minimum standard was adopted to obtain the value of the super parameter λ by tenfold cross-validation, where the optimal lambda resulted in 2 non-zero coefficients (n = 480). (**B**) Cross-validation for selecting tuning parameters. (**C**–**F**) Univariate (**C**,**E**) and multivariate (**D**,**F**) Cox regression analysis for overall survival (**C**,**D**) and progression free interval (**E**,**F**) of the relationship between *TBC1D8* and *TBC1D17* levels and clinicopathological parameters related to CRC prognosis in TCGA-COAD (n = 480). (**G**,**H**) Overall survival (OS) (**G**), Disease-specific survival (DSS) (**H**), and Progression-free interval (PFI) (**I**) based on median *TBC1D8* levels in TCGA-COAD (n = 480).

A total of 44 TBC structural domain family members were used for LASSO regression to identify robust markers for follow-up studies. Cross-validation was performed in ten rounds to prevent overfitting (Fig. 1A,B). Sequential Cox regression (both univariate and multivariate) indicated that *TBC1D8* was significantly correlated with CRC prognosis, identifying it as the research target (Fig. 1C–F). Finally, Kaplan–Meier survival curves showed that patients with higher *TBC1D8* levels had shorter OS and PFI (P < 0.05, Fig. 1G–H) and we did not observe an association between *TBC1D8* and DSS (P > 0.05, Figure S1). These results suggested that *TBC1D8* is closely associated with the progression of CRC and had potential research value.

### Analysis of TBC1D8 expression

**Figure 2:**
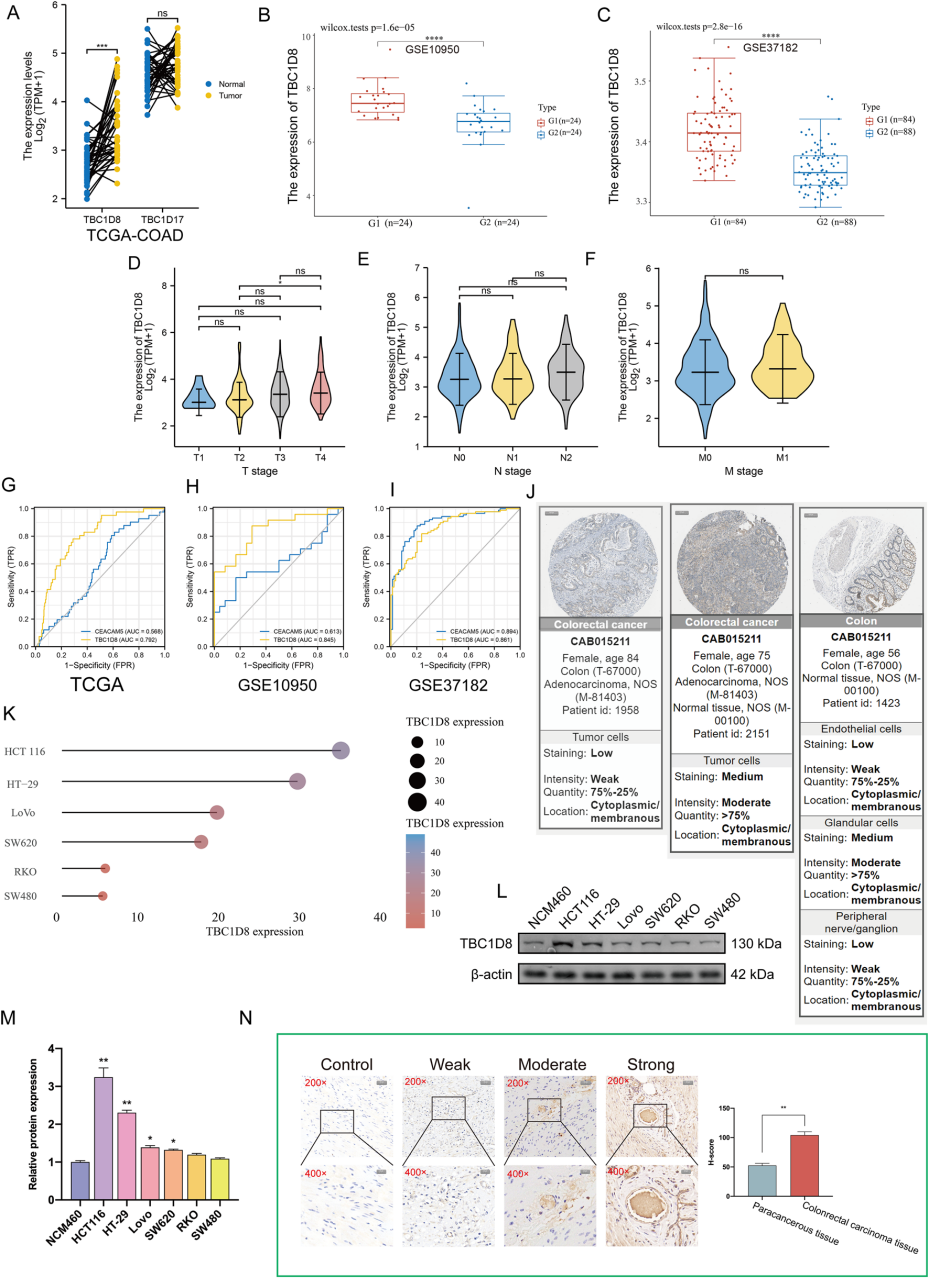
*TBC1D8* levels in colorectal cancer (CRC) and their association with clinicopathological parameters. (**A**) Expression level of *TBC1D8* between CRC tissues and paired paracancerous tissue in the TCGA-COAD. Wilcoxon test was performed (n = 41). (**B**,**C**) The differences in *TBC1D8* gene expression between CRC cases and normal controls in GSE10950 (n = 48) (**B**) and GSE37182 (n = 172) (**C**). Wilcoxon test was performed. (**D**–**F**) Association of *TBC1D8* mRNA levels with (**D**), T stage (n = 477) (**E**), N stage (n = 478) (**F**), and M (n = 411) stage in CRC patients based on TCGA-COAD. (**G**–**I**) Diagnostic Operating Characteristic (ROC) curves based on TBC1D8 and Carcino embryonic antigen (CEA) levels [**H**, TCGA (n = 521); **I**, GSE10950 (n = 48); **J**, GSE37182 (n = 172)]. (**J**) Immunohistochemical (IHC) analysis of TBC1D8 in CRC tissues based on Human Protein Atlas (HPA) (Magnification, × 2, scale bars = 500 μm). (**K**–**M**) *TBC1D8* expression in different CRC cell lines based on (**K**) the Cancer Cell Line Encyclopedia (CCLE) and (**L**,**M**) western blot. One-way ANOVA was conducted. Data in the bar chart are the means ± SME from three independent experiments. *P < 0.05. (**N**) Quantifications of TBC1D8 IHC staining based on our own samples (n = 30) show the differential expression between paracancerous and colonrectal carcinoma tissue. The t-test was performed (Magnification × 200, scale bars = 50 μm; Magnification ×  × 400, scale bars = 20 μm). (NS: non-significant, *P < 0.05, **P < 0.01, ***P < 0.001, ****P < 0.0001).

Data from TCGA-COAD indicated higher levels of *TBC1D8* transcription in CRC tissue (Fig. 2A). Moreover, information from the GSE10950 and GSE37182 datasets also showed higher *TBC1D8* levels in CRC compared to paracancerous tissues (Fig. 2B,C). It was found that there was no significant link between *TBC1D8* levels and TNM staging (Fig. 2D–F). Receiver operating characteristic (ROC) curves measured the ability of T*BC1D8* to differentiate CRC and normal tissue, with carcinoembryonic antigen (CEA) used as a positive reference. The areas under the curves are shown in Fig. 2G–I. The Human Protein Atlas (HPA) revealed that TBC1D8 was mostly cytoplasmic or membrane-associated (Fig. 2J). The results of the CCLE database are shown in Fig. 2K and the transcriptome expression levels of *TBC1D8* at cell level were, in descending order (HT-29, HCT 116, LoVo, SW620, RKO and SW480). The western blotting, shown in Fig. 2L,M, yielded consistent results. Subsequently, IHC demonstrated overexpression of TBC1D8 in CRC tissues compared to healthy tissues. We used our own CRC samples to detect TBC1D8 protein expression. The mean H-SCOREs for TBC1D8 in CRC and paracancerous tissues were 104.17 ± 5.79 and 52.58 ± 3.54, respectively (Fig. 2N). Results strongly indicated that an imbalance in the *TCB1D8* gene expression can cause CRC.

### Prediction of TBC1D8 functions and interactions

**Figure 3:**
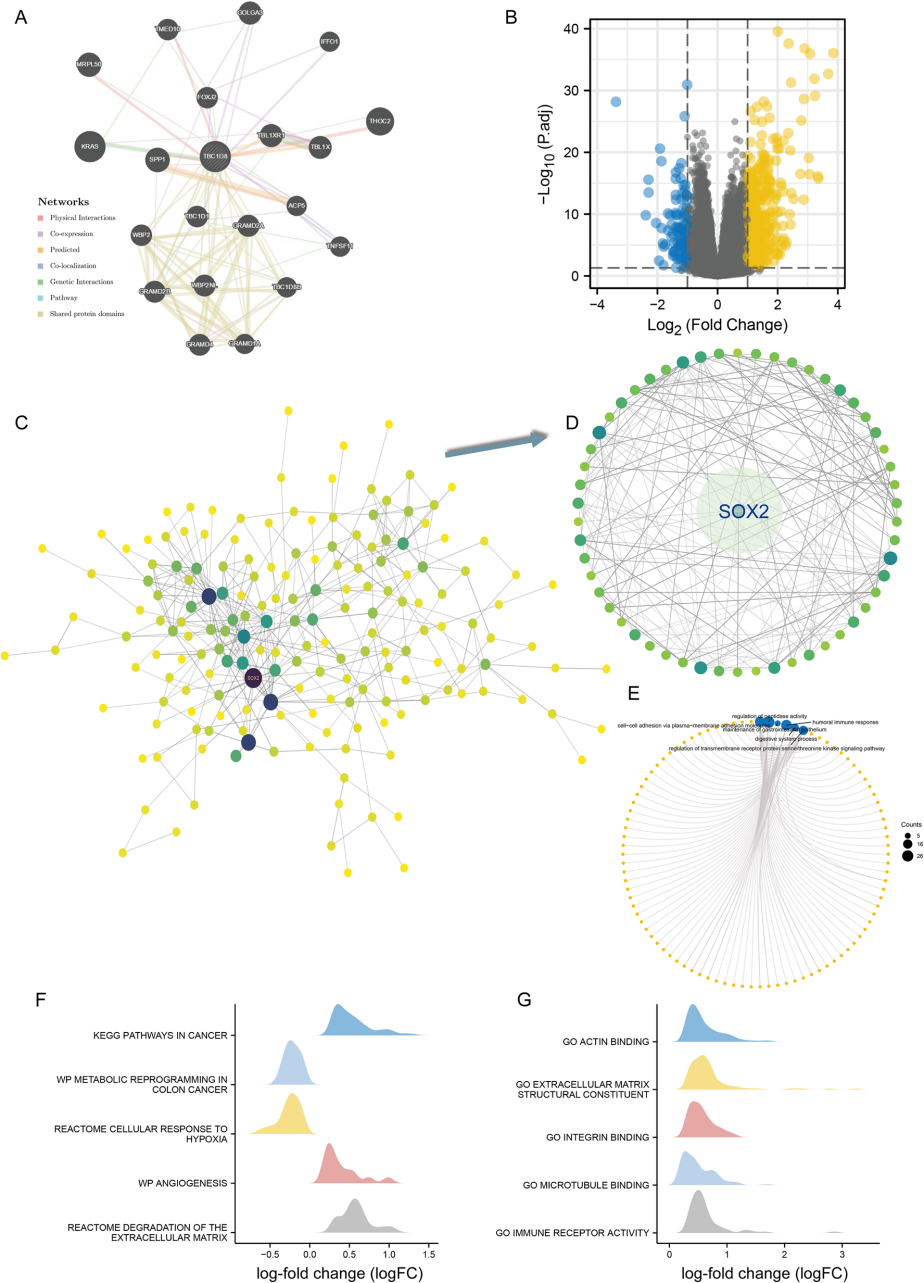
Protein–protein interaction (PPI) network and enrichment analysis. (**A**) *TBC1D8* with neighboring genes showing physical interactions, co-expression, co-localization, predicted common pathways, genetic interactions, and common protein domains based on GENEMANIA database. Each node represents a gene. The node size represents the strength of interactions, and the line color represents the types of interactions. (**B**) Volcano plot of Differentially expressed genes (DEGs) induced by alterations in *TBC1D8* levels. Yellow dots, upregulation; blue dots, downregulation; abscissa, expression differences (log2 fold change); ordinate, significance of differences (− log10 padj). (**C**) Cytoscape was conducted to visualized the network of *TBC1D8* and significantly correlated genes. Darker color and larger size indicate higher degree. (**D**) The hub module with the highest scores analyzed by Molecular Complex Detection (MCODE). Darker color and larger size indicate higher degree. (**E**) The “clusterProfiler” R package was used for the Gene ontology (GO) _biological_process (BP) enrichment analysis based on the module. (**F**,**G**) Gene Set Enrichment Analysis (GSEA) for *TBC1D8.* (**F**) The enriched gene sets in KEGG and Reactome collection by the high *TBC1D8* expression sample. (**G**) The enriched gene sets in GO collection by the high *TBC1D8* expression sample. Only gene sets with Normal P < 0.05 and false discovery rate (FDR) < 0.1 were considered significant and displayed in the plot (The x axis represents the distribution of log-fold change (logFC) corresponding to the core molecules in each gene set).

The network linking neighboring genes with *TBC1D8* was compiled by GeneMANIA. This shows various relationships, including interactions, colocalization, and common pathways (Fig. 3A). The genes co-expressed with *TBC1D8* in TCGA-COAD were examined using R "limma" (Fig. 3B). In all, 521 differentially expressed genes (DEGs) were used for the protein–protein interaction network (PPI) assembly. In the network, *SOX2* has the highest score as a hub gene based on cytoHubba (Fig. 3C). Enrichment analysis based on *SOX2* as the core sub-network showed that the network may involve "regulation of peptidase activity", "cell–cell adhesion via plasma-membrane adhesion molecules", "humoral immune response", "maintenance of gastrointestinal epithelium", "digestive system process", and “regulation of transmembrane receptor protein serine/threonine kinase signaling pathway” (Fig. 3D,E). GSEA for the whole PPI showed that *TBC1D8* may be associated with hypoxia, angiogenesis, and matrix degradation (Fig. 3F,G). These findings offered evidence regarding the relationship between *TBC1D8* and cancer-related signaling pathway. This led us to perform a more in-depth molecular mechanism study.

### Gene Set Variation Analysis (GSVA) based on TBC1D8 expression

**Figure 4:**
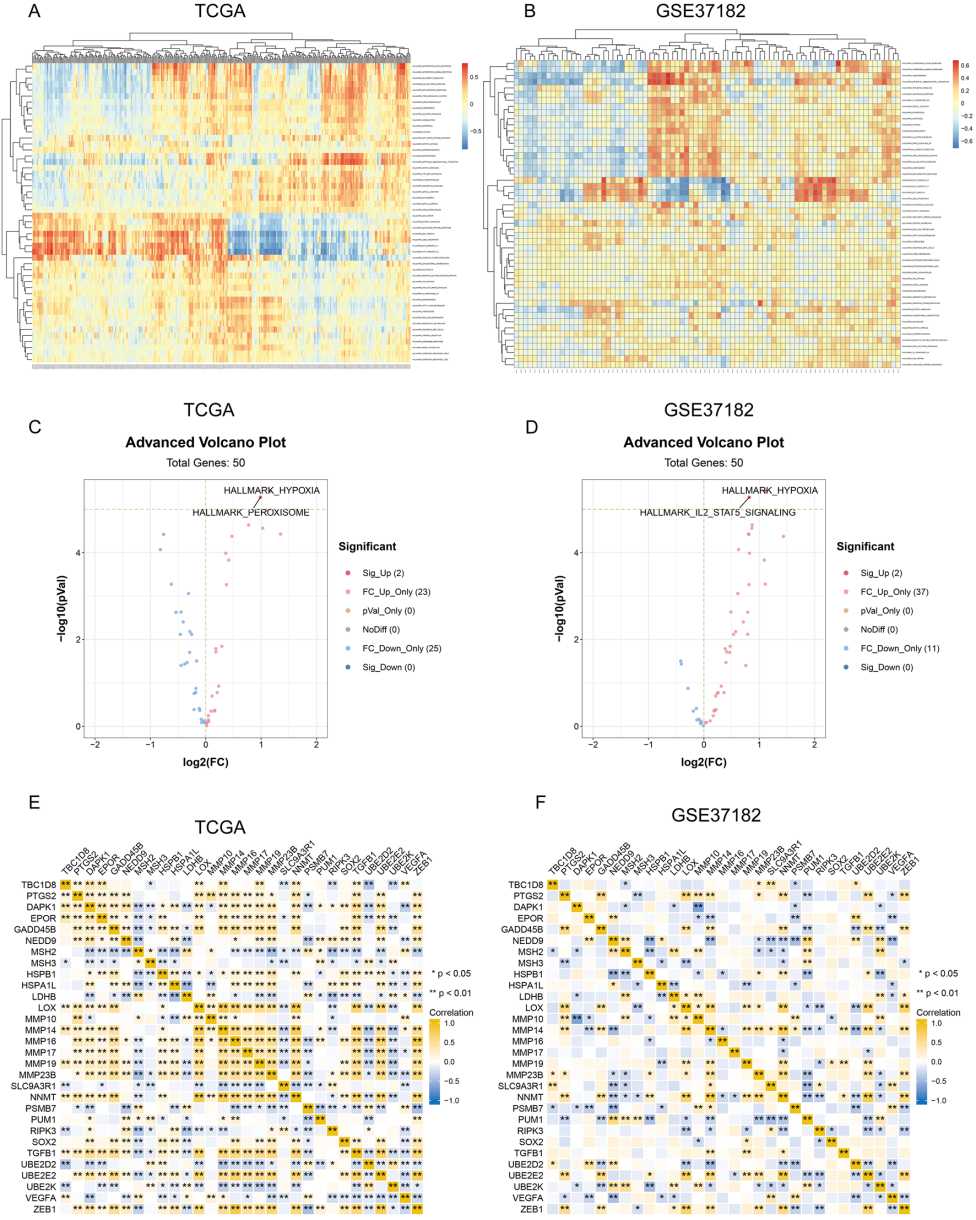
The differentially enriched genesets correlated with *TBC1D8* expression were studied through the gene set variation analysis (GSVA) algorithm based on TCGA and Gene Expression Omnibus (GEO) datasets. (**A**,**B**) Heatmap displaying the hierarchical clustering of enrichment scores obtained through Gene set variation analysis (GSVA) based on the enrichment degree profile of the datasets. (**A**) TCGA; (**B**) GSE37182. (**C**,**D**) Volcano plot of the differentially enriched genesets in CRC patients with different expression of *TBC1D8* in (TCGA) and (GSE37182) dataset was analyzed by GSVA by “limma” R package (**C** TCGA, n = 480, **D** GSE37182, n = 84). Blue nodes indicate downregulation, red indicates upregulation. (**E**,**F**) Relationship between *TBC1D8* and hypoxia-related genes based on TCGA (n = 480) (**E**) and GSE37182 (n = 84) (**F**). Yellow indicates a positive and blue indicates a negative relationship; darker color shows stronger correlation. (*P < 0.05, **P < 0.01).

The above results suggested that *TBC1D8* may be closely associated with the hypoxia phenotype, and here we performed a hallmark geneset-based GSVA to further analyze the possible involvement of *TBC1D8* in cancer-related pathways. The distribution of GSVA scores for the CRC samples in TCGA and GSE37182 is shown in Fig. 4A,B. The results of the differential score analysis based on the median TBC1D8 levels showed that “HYPOXIA” was active in the high-*TBC1D8* group, in both cohorts (P < 0.05) (Fig. 4C,D). The relationships between *TBC1D8* and hypoxia-associated genes were also examined (Fig. 4E,F). This further implied that *TBC1D8* play an important role in the adaptation to hypoxia.

### Single-cell level analysis of TBC1D8

**Figure 5:**
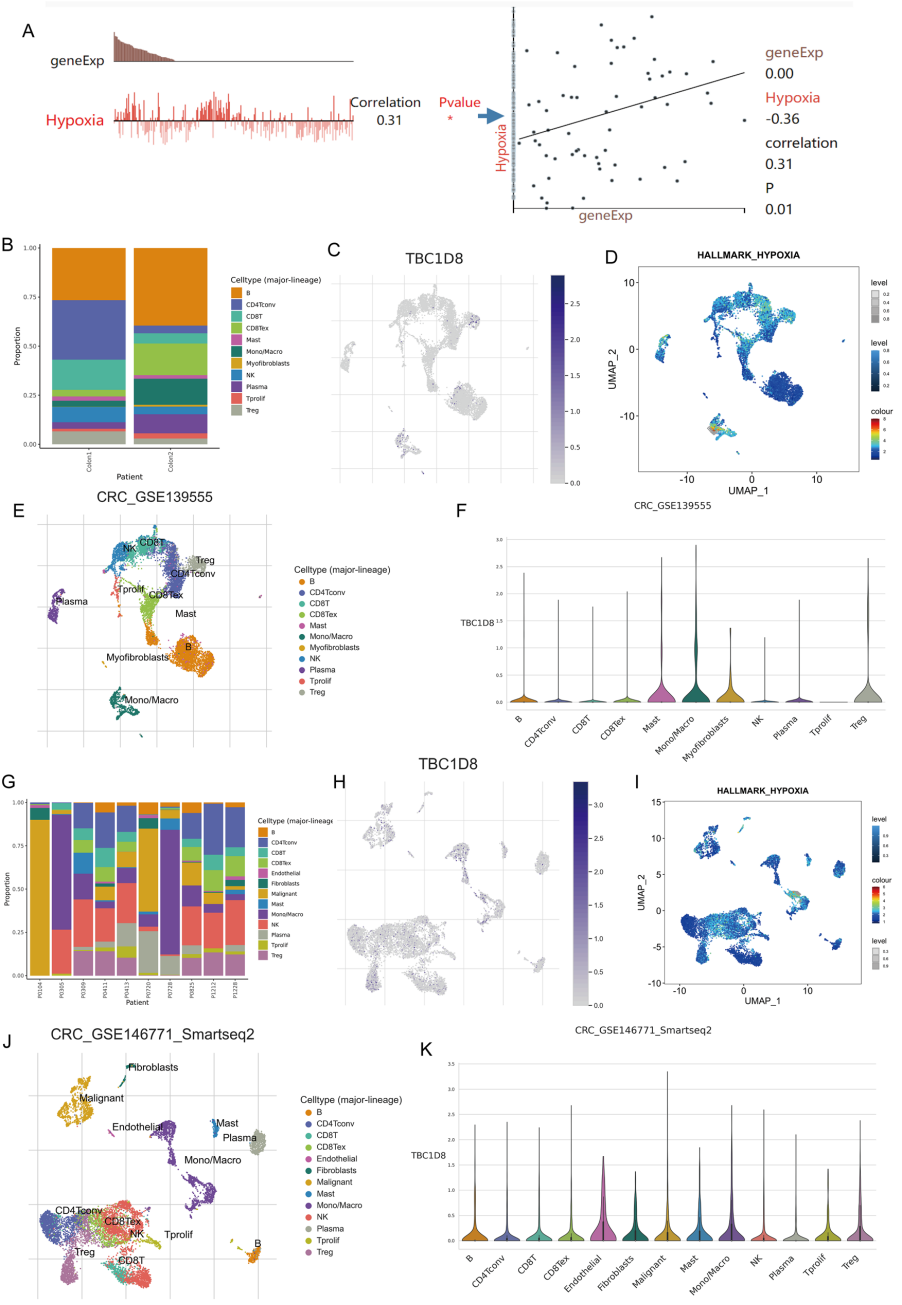
Single-cell analysis for *TBC1D8* based on Tumor Immune Single-cell Hub (TISCH) database*.* (**A**) Functional relevance of *TBC1D8* in patients with CRC. (**B**–**K**) (**B**,**G**) Cellular components based on (**B**) GSE139555 and (**G**) GSE146771. (**C**,**H**) Uniform manifold approximation and projection (UMAP) plots illustrating the expression of *TBC1D8* clusters based on (**C**) GSE139555 and (**H**) GSE146771. (**D**,**I**) Enrichment scores of genes from the Hallmark hypoxia geneset in individual cells, from gene set variation analysis based on (**D**) GSE139555 and (**I**) GSE146771. (**E**,**J**) UMAP plots showing the CRC cell landscape. Different cell types after quality control, dimensionality reduction, and clustering based on (**E**) GSE139555 and (**H**) GSE146771. (**F**,**K**) Violin plots for CRC cell cluster marker genes and *TBC1D8* in different cell types based on (**F**) GSE139555 and (**K**) GSE146771.

Herein, we sought to further search for an association between *TBC1D8* and hypoxia through single-cell datasets. The CancerSEA database found that *TBC1D8* levels were positively correlated with hypoxia (R = 0.31, P < 0.05) (Fig. 5A). The tumour microenvironment (TME) consists of cancer-associated fibroblasts (caf), myofibroblasts, immune cells and other components. to further examine the relationship between *TBC1D8* expression distribution and hypoxia, we explored the CRC single-cell datasets GSE139555 and GSE146771. Figure 5B,G show the cellular components of the two datasets, respectively. We observed that cells expressing TBC1D8 were enriched for hypoxia-associated genes (Fig. 5C,D,H,I) and found that TBC1D8 was expressed in both immune and stromal cell single cell subpopulations (Fig. 5E,F,J,K).

### TBC1D8 can be induced by hypoxic conditions, thereby contributing to tumor proliferation and tumor stemness

**Figure 6:**
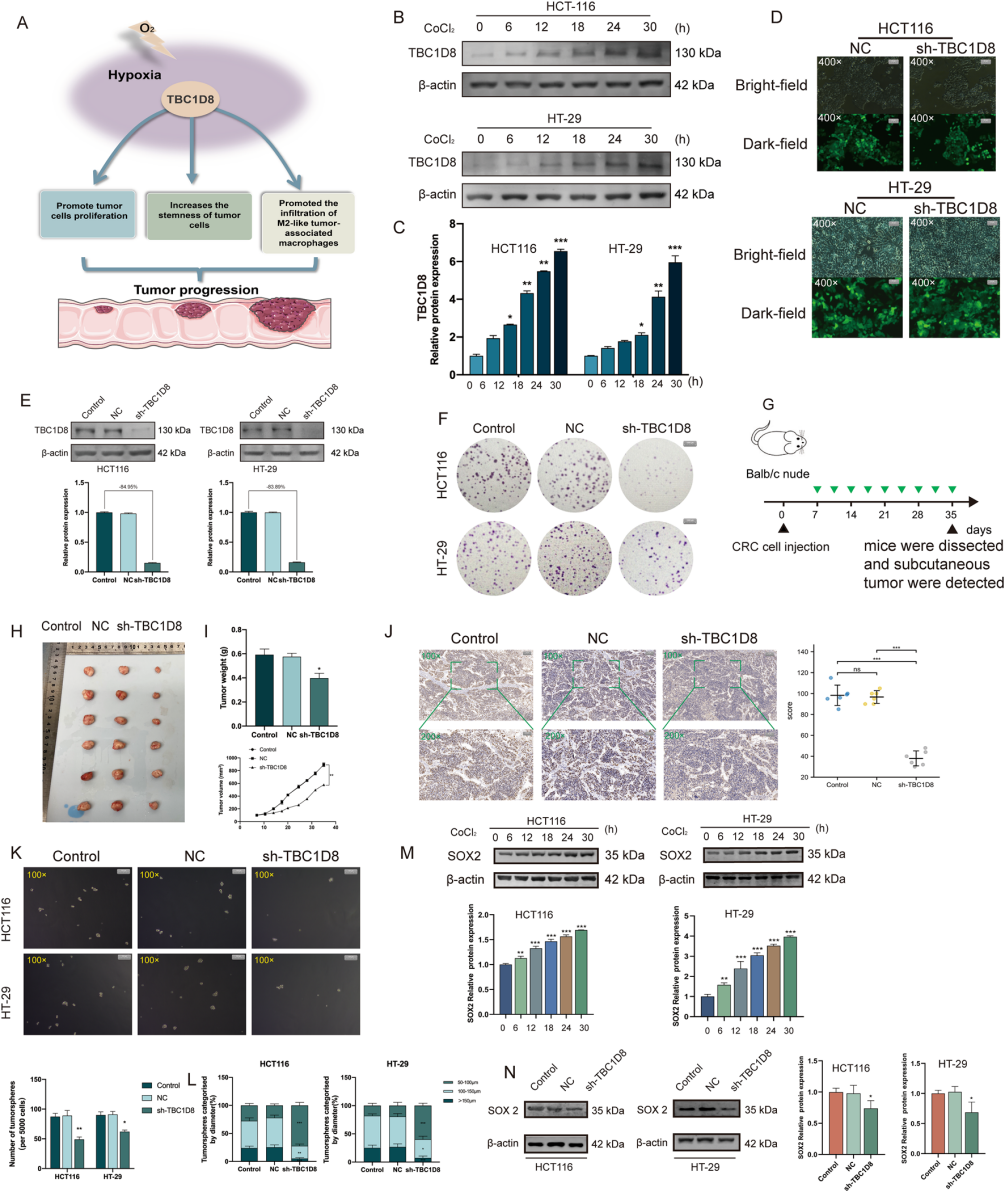
TBC1D8 can be induced by hypoxia conditions, thereby contributing to tumor proliferation and tumor stemness. (**A**) Schematic model of the possible role of *TBC1D8* in CRC. (**B**,**C**) Effects of CoCl2-induced hypoxia on *TBCD18* expression. (**D**,**E**) Transfection efficiencies (%) shown by Green fluorescent protein (GFP) expression and western blotting (Magnification, × 400, scale bars = 20 μm). (**F**) Clone formation capability of CRC cells transfected with NC and sh- TBC1D8 constructs, shown by colony formation determination (Magnification, × 1, scale bars = 1000 μm). (G) Schematic diagram of subcutaneous tumor models. (**H**) Xenograft mouse tumors (n = 6 mice per group). (**I**) Volumes of xenograft tumors measured twice a week and weights of xenograft tumors at completion of the study. (**J**) IHC staining of Ki-67 proteins in mouse xenograft tumor tissues (Magnification, × 100, scale bars = 100 μm; Magnification × 200, scale bars = 20 μm). All the IHC scores were repeated three times using a double-blind method.Statistical analysis All experiments were repeated at least three times, independently. (**K**) Sphere-forming assay of cells (magnification, × 100, scale bars = 100 μm). (**L**) The number of cell spheres and the sphere size were measured. All experiments were repeated at least three times, independently. (**M**) The relative expression level of SOX2 under hypoxic condition was detected using Western blot analysis (n = 3 replicates). (**N**) The relative expression level of SOX2 levels in CRC cells transfected with NC and sh-*TBCID8*, were examined using western blot analysis (n = 3 replicates). Data are means ± SEM *p < 0.05; **p < 0.01; ***p < 0.001. One-way ANOVA was conducted. All experiments were repeated at least three times, independently.

The enrichment, GSVA, and single-cell analysis results suggested that *TBC1D8* may be associated with hypoxia. Given that its Hub Gene is an indicator of *SOX2*, which is involved in the development and maintenance of stem-like properties in cancer cells, we considered that *TBC1D8* may also be associated with tumor cell stemness. A possible mechanism is proposed in Fig. 6A. Based on the above results we performed a preliminary experimental validation. To simulate neoplastic hypoxic microenvironmental conditions, we used CoCl_2_ to induce a hypoxic environment. It was found that TBC1D8 expression gradually increased with prolongation of the CoCl_2_ treatment (Fig. 6B,C). Western blot analysis was used to determine levels of hypoxia-inducible factor (HIF)-1α to confirm the induction of hypoxia (Figure S2). High levels of TBC1D8 were seen in HCT116 and HT-29 cells, so these were used for further study. Transfection efficiencies were confirmed by GFP intensity and western blotting (Fig. 6D,E). Silencing of *TBC1D8* reduced clone formation (Fig. 6F). The schematic diagram of subcutaneous tumor models is presented in Fig. 6G. Moreover, *TBC1D8* knockdown suppressed xenograft tumor growth in vivo (Fig. 6H,I). IHC staining of xenograft tumors revealed a significant decrease in cytosolic Ki-67 expression accompanied by knockdown of *TBC1D8* (Fig. 6J). Tumor sphere formation is documented to be linked with the stemness of cancer cells. Quantification of spheres showed that *TBC1D8* knockdown significantly reduced both the numbers (Fig. 6K) and sizes (Fig. 6L) of CRC cell tumor spheres. The expression levels of SOX2 were significantly increased following exposure to hypoxia compared with those under normoxic conditions (Fig. 6M). The expression levels of SOX2 were markedly downregulated following *TBC1D8* knockdown (Fig. 6N). This part of the results suggested that *TBC1D8* plays an important role in linking hypoxia and stem cell characteristics of CRC cells.

### Immune microenvironment analysis of TBC1D8

**Figure 7:**
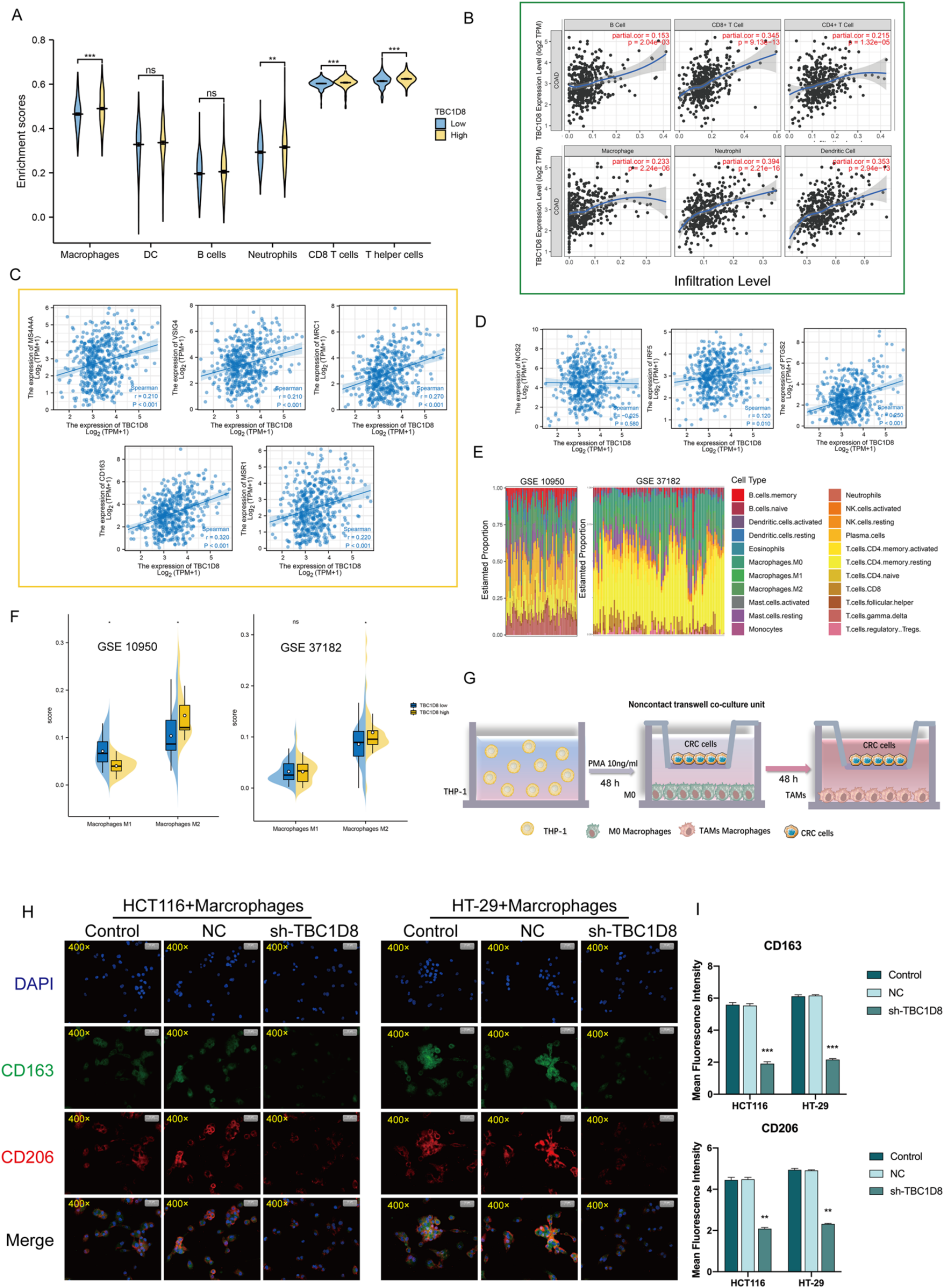
Immune analysis of *TBC1D8*. (**A**) Effects of high and low T*BC1D8* expression on immune cells. (**B**) Relationship between *TBC1D8* level and degree of immune infiltration, from TIMER. (**C**,**D**) Relationship between *TBC1D8* and levels of (**C**) M2 macrophage markers and (**D**) M1 macrophage markers. (**E**) Tumor-initiating cell proportions in CRC samples. from GSE10950 and GSE37182 data based on CIBERSORT. (**F**) Relationship between *TBC1D8* level and macrophage abundance based on CIBERSORT, from GSE10950 and GSE37182 data. (**G**) Diagram of co-culture system. (**H**) Double-immunofluorescence staining of M2 macrophage markers CD206 (red) and CD163 (green); nuclei are stained with DAPI (blue) (Magnification, × 400, scale bars = 20 μm) (n = 3 replicates). (**I**) Intensity of immunofluorescence (mean ± SEM) (n = 3 replicates). Data are means ± SEM *p < 0.05; **p < 0.01; ***p < 0.001.

In this section, we explored the relationship between immune infiltration and CRC pathogenesis. Relationships between *TBC1D8* and infiltration were examined with the EPIC algorithm. This showed that macrophages, neutrophils, CD8 T cells, and T helper cells were present at higher levels when *TBC1D8* was strongly expressed (Fig. 7A). TIMER was also used to explore potential correlations between *TBC1D8* levels and immune cell infiltration (Fig. 7B). There were positive correlations between *TBC1D8* and CD8 + T cells (R = 0.345, P = 9.13e−13), CD4 + T cells (R = 0.215, P = 1.32e−05), macrophages (R = 0.233, P = 2.24e−06), neutrophils (R = 0.394, P = 2.21e−16), dendritic cells (R = 0.353, P = 2.94e−13), and B cells (R = 0.153, P = 2.04e−03) levels.

We further analyzed the relationship between *TBC1D8* and macrophage polarization based on TCGA-COAD. *TBC1D8* was positively correlated with *MS4A4A* (R = 0.210, P < 0.001), *VSIG4* (R = 0.210, P < 0.001), *MRC1* (R = 0.270, P < 0.001), *CD163* (R = 0.320, P < 0.001), *MSR1* (R = 0.220, P < 0.001), *IRF5* (R = 0.120, P = 0.010), and *PTGS2* (R = 0.250, P < 0.001) but not with *NOS2* (P = 0.580) (Fig. 7C,D). Finally, we further found that M2 macrophages had a higher degree of infiltration in CRC patients in the *TBC1D8* high expression group using GSE10950 and GSE37182 (Fig. 7E,F). This series of results suggested the presence of a positive association between *TBC1D8* expression and M2 macrophage infiltration. To further investigate the influence of *TBC1D8* expression on M2 macrophage abundance in CRC, we established a tumor–macrophage co-culture model using a transwell non-contact co-culture unit (Fig. 7G). We observed that *TBC1D8* knockdown significantly down -regulated the surface markers of M2 tumor-associated macrophages (TAMs) (CD206 and CD163) in THP-1 macrophages (Fig. 7H,I). Our results suggested that TBC1D8 can exert immunosuppressive functions by promoting the proliferation of M2 macrophages.

### Genetic alteration analysis and DNA methylation of TBC1D8

**Figure 8:**
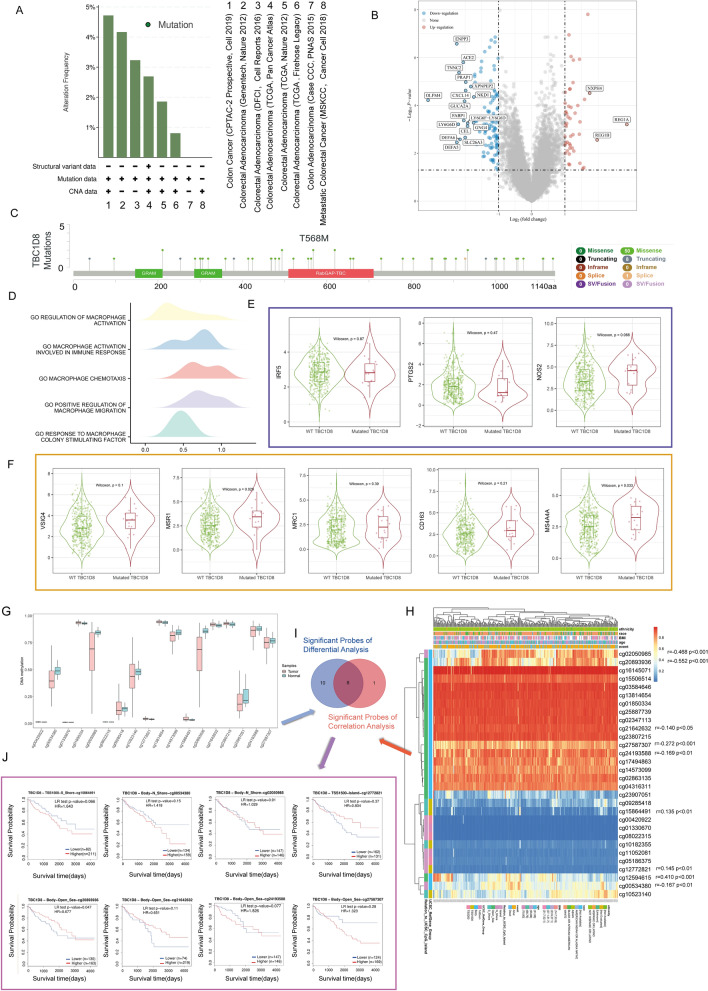
The genetic alteration of *TBC1D8* in CRC. (**A**) Frequencies of *TBC1D8* mutations and copy number alterations (CNA) in the 8 datasets shown on the right side. (**B**) Volcano map of genes showing differential expression after a change in *TBC1D8* (mutated and wild). Red dots, upregulated genes; blue dots, downregulated genes; abscissa, differences in gene expression (log2 fold change); and ordinate, significance of these differences (− log10 padj). (**C**) The mutation site profile of the *TBC1D8* gene is shown. (**D**) GSEA was used to determine the functions of differential gene sets between the mutated and wild groups based on GO. Only gene sets with Normal P < 0.05 and FDR < 0.1 were considered significant and displayed in the plot. The x axis represents the distribution of log-fold change (logFC) corresponding to the core molecules in each gene set. (**E**–**F**) Differential Analysis of *TBC1D8* (mutated and wild) with M1 (**E**) and M2 (**F**) macrophage markers. Wilcoxon-Mann–Whitney test was performed based on TIMER database. (**G**) The Differential Analysis of the *TBC1D8* probe methylation were indicated. (**H**) Waterfall plot of the methylation levels in the *TBC1D8* gene. The correlations between *TBC1D8* methylation or expression levels were also analyzed. (**I**) Venn diagram showing the intersection of (**G**,**H**). (**J**) Survival analysis based on the intersection methylation probes; P < 0.05 was considered statistically significant.

Here, we attempted to investigate the potential mechanism of *TBC1D8* in CRC in terms of mutation and DNA methylation. We first detected the mutation frequency in 8 groups of CRC cases through the cBioPortal database (Fig. 8A). Volcano map showing DEGs after a change in *TBC1D8* (mutated and wild) (Fig. 8B). Mutation site profile of the *TBC1D8* shown in Fig. 8C. After identifying the DEGs, we then performed GSEA to research the functional enrichment of these DEGs. The DEGs were enriched in “REGULATION OF MACROPHAGE ACTIVATION”, “MACROPHAGE ACTIVATION INVOLVED IN IMMUNE RESPONSE”, “MACROPHAGE CHEMOTAXIS”, “POSITIVE REGULATION OF MACROPHAGE MIGRATION”, “RESPONSE TO MACROPHAGE COLONY STIMULATING FACTOR” (Fig. 8D), which all point to macrophages. Subsequently, M1 and M2 macrophage markers were analyzed under *TBC1D8* (mutated and wild) status (Fig. 8E,F). Differential methylation status of *TBC1D8* between CRC tissues and normal tissues were analyzed. The statistically significant CG sites are shown in the Fig. 8G. Based on methylation data from TCGA-COAD, we established that the methylation values obtained from 9 methylation probes were significantly correlated with *TBC1D*8 expression levels (Fig. 8H). The intersection of CG sites in the above two results (Fig. 8I) were then used to perform a survival analysis and cg20893936 was identified to be related to CRC patients’ survival (Fig. 8J). These results suggested that *TBC1D8* gene DNA alternation may affect colorectal tumorigenesis.

## Discussion

CRC is a heterogeneous disease, which occurs and progresses in a complex microenvironment^41^. Hypoxia is an important feature of the colorectal cancer microenvironment, and at the same time, hypoxia makes tumor signaling pathways more intricate^42,43^. In this complex molecular network, some genes are upregulated under hypoxic conditions and can contribute to the malignant biological behavior of tumor cells through multiple signaling cascades^44,45^. This also means that there are more opportunities to find effective targets for action. With the support of computational disciplines such as bioinformatics and systems biology, our group has used several publicly available online databases to initially identify an oncogene that has not been previously mentioned in colorectal cancer. This provides a potentially interesting direction for the development of therapeutic, targeted drugs for colorectal cancer.

Several studies have stated that a dysregulated TBC family expression is involved in various human diseases such as cancer, obesity and X-linked early-onset nephrotic syndrome and so on^46–49^. There are few reports on TBC1 domain family members in tumor, but the available studies suggest that it mostly functions as an oncogene in tumors. *TBC1D3* overexpression inhibits the ubiquitination and degradation of Epidermal growth factor receptor (EGFR), thereby enhancing the EGFR signaling pathway and promoting cell proliferation^50^. *TBC1D15* has been identified as a powerful oncogene in hepatocellular carcinoma, and the mechanisms involved include promotion of Tumor-initiating-stem-like cells self-renewal and p53 deletion^51^. In addition, *TBC1D23* has been suggested to be a powerful promoter of tumor cell proliferation, migration, and invasion in non-small cell lung cancer^52^. In conclusion, most of the available studies reveal a possible fact that TBC1 domain family members can participate in malignant cell transformation or metastasis by regulating Rab proteins. However, to date, few studies have been reported on its involvement in colorectal cancer.

In this study, we first analyze the LASSO regression based on TCGA-COAD, and showed that *TBC1D8* and *TBC1D17* were potentially valuable research targets. While it has been reported that *TBC1D8* can regulated metabolic reprogramming to drive ovarian cancer progression, little research has been reported on *TBC1D17* in cancer. *TBC1D17* is currently identified as a mitochondria-localized RAB7A GTPase-activating protein (GAP) that helps maintain mitochondrial homeostasis by regulating RAB7A activity and limiting mitophagy. And a work by Xi Sheng Rao et al. demonstrated that *TBC1D17* can act as a molecular bridge linking AMPK and Rab5 to play the role of an GTPase activating protein to regulate glucose homeostasis. Further Cox analysis incorporating TNM staging suggested that *TBC1D8* may be an independent prognostic factor for CRC, and therefore we selected *TBC1D8* as our study target. The results of three independent CRC datasets showed significantly elevated transcription of *TBC1D8* in CRC tissues and, the HPA database and our samples further validated the overexpression of TBC1D8 in colorectal cancer at the protein level.

In order to explore the possible biological function of *TBC1D8*, we identified 578 significantly related genes based on TCGA-COAD and performed a functional enrichment analysis. The results showed that *TBC1D8* and its related genes are significantly associated with many related to the tumor microenvironment, including “ANGIOGENESIS”, “DEGRADATION OF THE EXTRACELLULAR MATRIX”, “ACTIN BINDING”, and “INTEGRIN BINDING”. All these processes are important components of the tumor microenvironment, and they can interact to further promote tumor growth and metastasis. It is worth noting that we have observed that TBC1D8 may also be related to the hypoxic phenotype, and by analyzing the TBC1D8-based PPI, we found that SOX2, a hypoxia-related indicator, was the highest-scoring hub gene. It has been well documented that *SOX2* can promote tumor proliferation and invasion and can regulate tumor cell stemness in a hypoxic microenvironment through a variety of pathways^53^. Cell sphere formation capacity have been used as read-out for tumor stemness^54^.Previous studies have reported a less differentiated phenotype and/or an increase in stemness induced by hypoxia in Various tumors^55–57^. In the present study, it was shown that TBC1D8 knockdown potentiated sphere-forming capacity in CRC cells. However, the correlation between *SOX2* and *TBC1D8* was poor. This implies that *TBC1D8* may be performing a similar biological function to *SOX2* although through different mechanisms. On the one hand, hypoxia leads to a transcriptional program promoting basement membrane degradation, while increasing the ab initio synthesis of protofibrillar collagen as a physical pathway of tumor invasion^58,59^; on the other hand, hypoxia is a potent promoter of tumor-associated angiogenesis^60^, which was significantly activated in the high *TBC1D8* expression group in the GSVA. Single-cell level analysis also suggested that cells expressing *TBC1D8* were enriched for hypoxia-associated genes. We calculated the correlation between *TBC1D8* and hypoxia-related genes, however, no consistent results were observed in two independent datasets, which may be due to heterogeneity between samples. We then used a cellular model of hypoxia to verify the upregulation of *TBC1D8* under hypoxic conditions. Based on these results, we subsequently verified the biological functions of *TBC1D8* in CRC cells. This showed that blocking *TBC1D8* expression inhibited CRC cell proliferation, decreased clonogenic formation, invasion, and stemness in vitro. In addition, growth factor secretion induced by the hypoxic tumor environment also promotes macrophage aggregation, and macrophages are sensitive to hypoxia and alter their gene expression accordingly. Recent studies have found that macrophages in hypoxic regions induce fibrosis by growth factor production, thereby attracting both additional macrophages and mesenchymal cells^61,62^. Based on this observation, we examined the link between *TBC1D8* and macrophage infiltration, finding that CRC patients with high *TBC1D8* expression tended to have higher levels of M2 macrophage infiltration. Subsequent results also supported this conclusion. Because hypoxia broadly affects molecular events involved in cancer progression, aggressiveness, and treatment resistance, targeting hypoxia is an attractive approach for solid cancer treatment^63^. However, in practice, it is difficult to quantify hypoxia specifically, while targeting specific hypoxia-related targets is a feasible option. On the one hand, hypoxia can help tumor cells maintain stem cell-like characteristics to enhance their aggressiveness^64^, while on the other hand, immunosuppressive cells, including M2 macrophages^65,66^, accumulate in hypoxic regions of the tumor where they promote tumor progression and activate immune tolerance mechanisms that enable cancer cells to evade host immune surveillance^67,68^. Thus, targeting the hypoxia-related molecular signaling cascade network not only helps to improve tumor resistance to conventional chemotherapy, but also increases the probability that tumor cells will be recognized and killed by the body's immune system.

Altered genomic status is strongly associated with CRC carcinogenesis^69^. We observed that that *TBC1D8* has a reasonable chance of being mutated in CRC. Functional enrichment suggested that *TBC1D8* mutations may be associated with macrophage activation, and further calculations also showed that *MSR1* and *MS4A4A* tend to be more strongly expressed in patients with mutated *TBC1D8* in CRC. MSR1 and MS4A4A proteins have been shown to be associated with the activation of both tumor-associated and M2 macrophages^70^. Accordingly, we hypothesize that mutant *TBC1D8* may be more able to activate immunosuppressive macrophages. Considering that DNA methylation is the most important method of epigenetic modification, we also observed that the degree of methylation of cg20893936 may correlate with the prognosis of CRC patients and can serve as a diagnostic biomarker. Further investigation into the question of how methylation of different sites in *TBC1D8* affects the outcomes of CRC patients is warranted.

In conclusion, in this study, we found that *TBC1D8* is a potential marker for both CRC diagnosis and prognosis. *TBC1D8* may function as an oncogene triggered by hypoxia, a result that could lay the foundation for subsequent studies and experimental verification.

## Supplementary Information

Supplementary Information.
