# Supplementary material for: An analysis of the significance of the Tre2/Bub2/CDC 16 (TBC) domain protein family 8 in colorectal cancer
Source: Sci Rep. 2022 Aug 2;12:13245. doi: 10.1038/s41598-022-15629-1 (PMC9345998; doi:10.1038/s41598-022-15629-1)

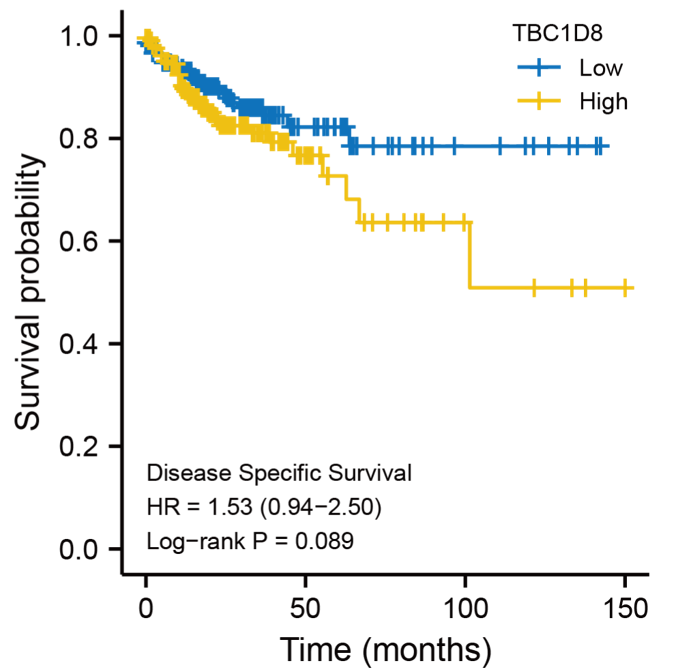


Figure S1 Disease-free survival (DFS) analyses according to the expression level of the TBC1D8 gene were performed using CRC cases in the TCGA-COAD cohort.


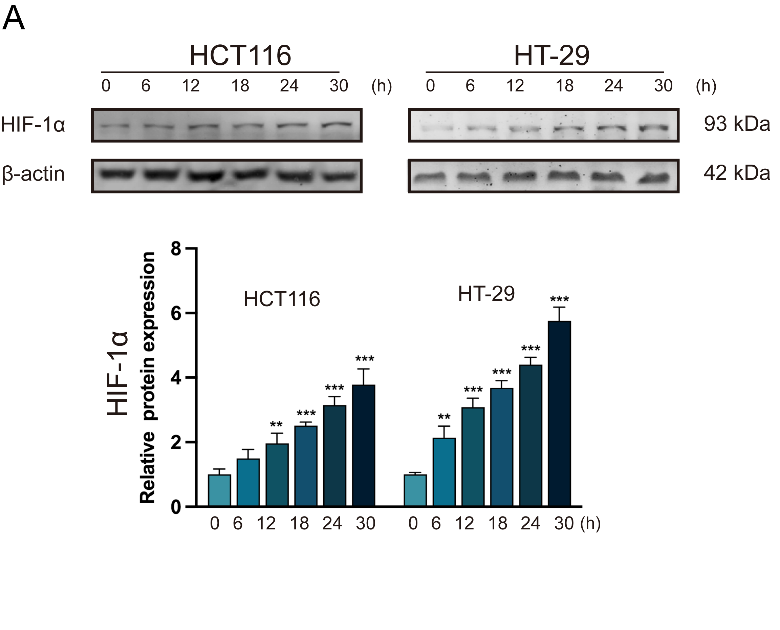


Figure S2 （A）Western blot analysis of HIF-a was used to confirm successful induction of hypoxia. Data in the bar chart are the means ± SME from three independent experiments. *P < 0.05.

## Supplementary Table 1

| **Antibodies and Reagents** | **Manufacturer, Country, Batch number** | **Concentration** |
| --- | --- | --- |
| Roswell Park Memorial Institute 1640 (RPMI-1640) | Gibco, USA, Lot: 8121248 | - |
| Dulbecco’s modified Eagle’s medium (DMEM) | Gibco, USA, Lot: 8121032 | - |
| Fetal bovine serum (FBS) | Gibco, USA, Lot: 42F1376K | - |
| Penicillin/streptomycin | Gibco, USA, Lot: 2321125 |  |
| GMyc-PCR Mycoplasma Test Kit | YEASEN, China, Lot: G5110070 |  |
| Plasmid vectors for TBC1D8 | GeneChem, China, Lot: GIEE0312855 |  |
| Puromycin | Beyotime Biotechnology, China, Lot: 041321210517 | 1.5 μg/mL |
| Lipofectamine 3000 | Invitrogen, USA, Lot: 2395298 | - |
| Opti-MEM | Gibco, USA, Lot: 2276923 | - |
| Cobalt (II) chloride, anhydrous, 97% | Thermo Fisher Scientific, USA, Lot: B22031 | 300 µmol/L |
| Anti-TBC1D8 Antibody | Santa Cruz Biotechnology, USA, Lot: sc-376637 | WB: 1:1000 |
| HIF1A Polyclonal Antibody | Thermo Fisher Scientific, USA, Lot: VL3162393 | WB: 1:1000 |
| Anti-β-actin Antibody | Invitrogen, USA, Lot: RI2265993 | WB: 1:5000 |
| Recombinant Human EGF  Animal-Free manufactured | Peprotech, USA. Lot: 111908 | 20ng/ml |
| Recombinant Human FGF-basic (154 a.a.) | Peprotech, USA. Lot: 0820AFC05 | 20ng/ml |
| B27 supplement | Gibco, USA, Lot:2226816 | 2% |
| anti-CD163 polyclonal antibodies | Proteintech, China, Lot: 00091171 | 1:1000 |
| anti-CD206 polyclonal antibodies | Proteintech, China, Lot: 00089604 | 1:1000 |
| phorbol 12-myristate 13-acetate (PMA) | Sigma-Aldrich, USA. Lot: SLBX8899 | 10 ng/mL |
| Alexa Fluor 488 AffiniPure goat anti-mouse IgG (H+L) | FcMACS, China, Lot: 136908 | 1:2000 |
| Goat anti-mouse IgG (H+L) CoraLite594 | Proteintech, China, Lot: 20000154 | 1:1000 |
| Goat anti-rabbit IgG (H+L) R-PE conjugate | Proteintech, China, Lot: 20000129 | 1:1000 |

## TBC1D8 RNAi preparation

**Gene information:**

| **Gene symbol** | **GenBank_ID** |
| --- | --- |
| TBC1D8 | NM_032609 |

**Target information:**

| **ID** | **Target equence information：** | **gene start position** | **GC percentage (%)** |
| --- | --- | --- | --- |
| TBC1D8-RNAi(87293-2) | gcTGCCTCGATATTATGTATA | 2839 | 31.58 |
| TBC1D8-RNAi(87294-1) | gaACGTGCTTCGAGTCGTTAT | 2531 | 47.37 |
| TBC1D8-RNAi(87295-1) | cgAAACGGGAATTGCTGCTTT | 1829 | 42.11 |

**shRNA Sequence：**

| **ID** | **5’** | **stem** | **loop** | **stem** | **3’** |
| --- | --- | --- | --- | --- | --- |
| TBC1D8-RNAi(87293-2)-a | Ccgg | gcTGCCTCGATATTATGTATA | CTCGAG | TATACATAATATCGAGGCAGC | TTTTTg |
| TBC1D8-RNAi(87293-2)-b | aattcaaaaa | gcTGCCTCGATATTATGTATA | CTCGAG | TATACATAATATCGAGGCAGC |  |
| TBC1D8-RNAi(87294-1)-a | Ccgg | gaACGTGCTTCGAGTCGTTAT | CTCGAG | ATAACGACTCGAAGCACGTTC | TTTTTg |
| TBC1D8-RNAi(87294-1)-b | aattcaaaaa | gaACGTGCTTCGAGTCGTTAT | CTCGAG | ATAACGACTCGAAGCACGTTC |  |
| TBC1D8-RNAi(87295-1)-a | Ccgg | cgAAACGGGAATTGCTGCTTT | CTCGAG | AAAGCAGCAATTCCCGTTTCG | TTTTTg |
| TBC1D8-RNAi(87295-1)-b | aattcaaaaa | cgAAACGGGAATTGCTGCTTT | CTCGAG | AAAGCAGCAATTCCCGTTTCG |  |

**Plasmid name ：**GV493

**Element’s order：**hU6-MCS-CBh-gcGFP-IRES-puromycin

**Negative control number：**CON313

**
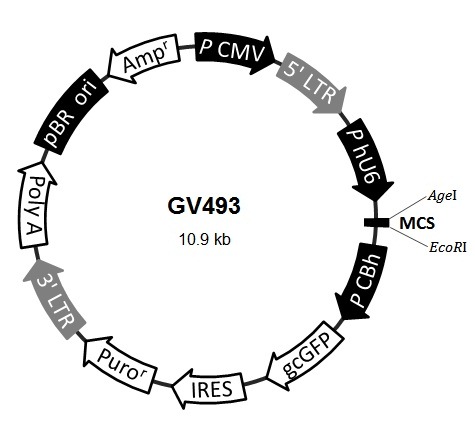
Negative control insert sequence：**TTCTCCGAACGTGTCACGT

## Phorbol-12-myristate 13-acetate (PMA) induction scheme selection

The THP-1 cell line is a human leukemia cell-derived monocyte line that is capable of acquiring phenotypic and functional characteristics similar to those of primary macrophages upon PMA stimulation[1, 2]. It is currently the most widely used in vitro model of human-derived macrophages. It has been proven that 5 ng/mL PMA treatment can cause THP-1 monocytes to differentiate into macrophages. In addition, many researchers believe that the higher the PMA concentration, the easier it is for differentiated macrophages to transform into the M1 subtype[3]. Therefore, a low-concentration induction protocol was chosen for this study. Due to various inter-laboratory variations, initial induction with 5 ng/mL PMA for 48 h resulted in only about 60% of the THP-1 cells adhering to the wall. Subsequently, after many adjustments, 10 ng/mL for 48 h was selected as the induction model, as 95% of THP-1 cells differentiated and adhered to the wall under this treatment condition.THP-1 cells in logarithmic growth phase were centrifuged and resuspended in RPMI-1640 medium; after adding 10 ng/mL PMA to induce 48 hour, THP-1 transformed from suspension growth to wall growth, from round to irregular shape, further increased in size, cell pulp was loosened, cell nucleus enlarged obviously, a large number of obvious organelles were visible, and a small amount of protrusion around the cytosol was visible.


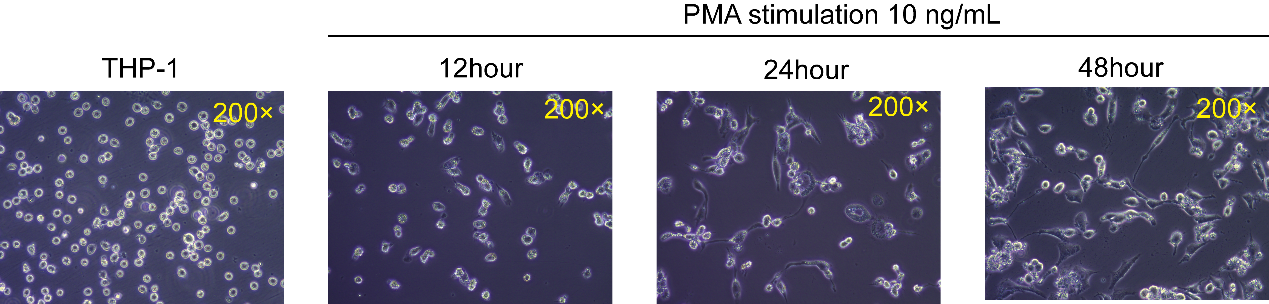


Morphological change of M0 macrophages derived from THP-1 monocytes. It was studied under the Olympus BX50 light microscope (200× magnification)

# Reference

1. Tsuchiya, S., et al., *Induction of maturation in cultured human monocytic leukemia cells by a phorbol diester.* Cancer Res, 1982. **42**(4): p. 1530-6.

2. Genin, M., et al., *M1 and M2 macrophages derived from THP-1 cells differentially modulate the response of cancer cells to etoposide.* BMC Cancer, 2015. **15**: p. 577.

3. Chanput, W., et al., *Characterization of polarized THP-1 macrophages and polarizing ability of LPS and food compounds.* Food Funct, 2013. **4**(2): p. 266-76.

## original uncropped western blots


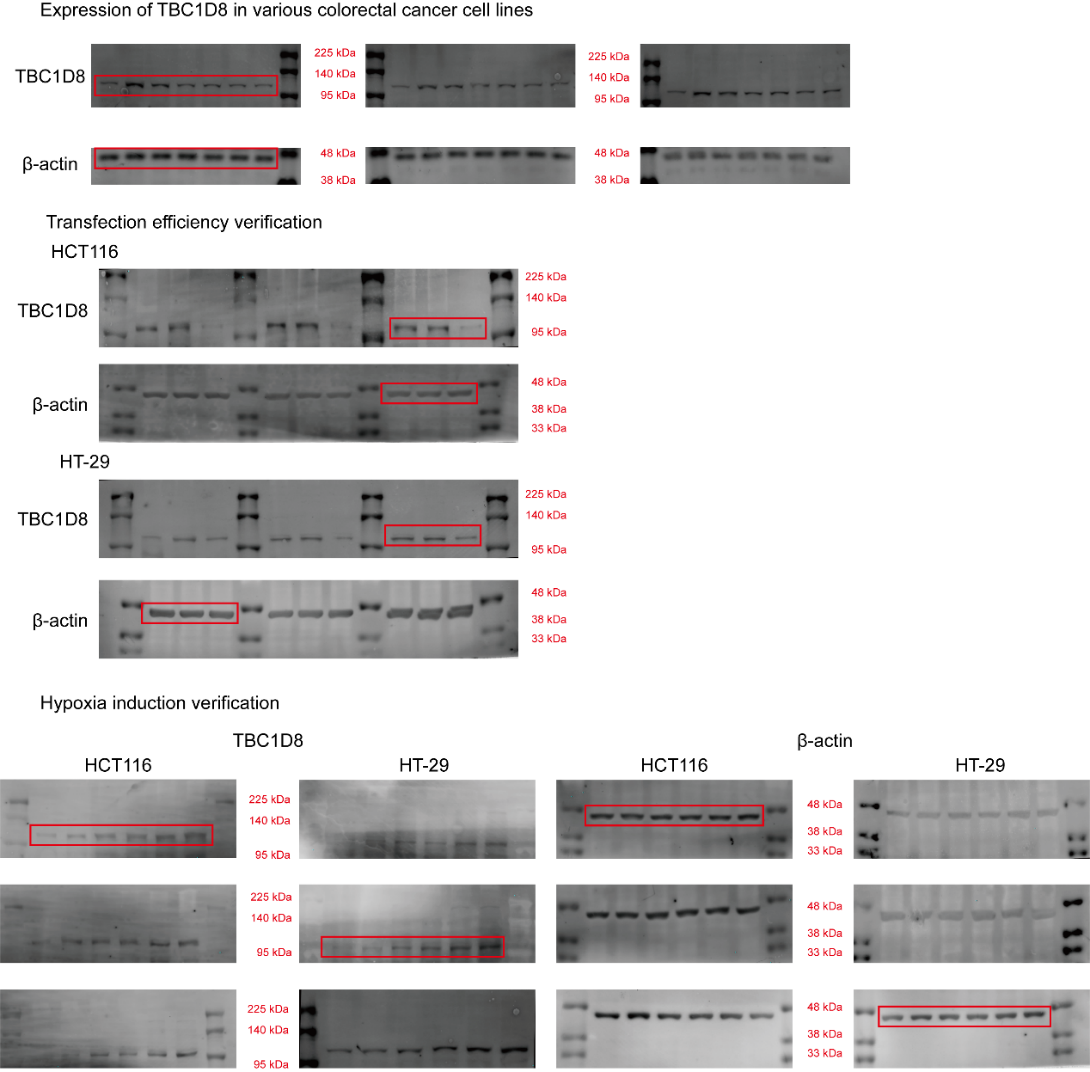


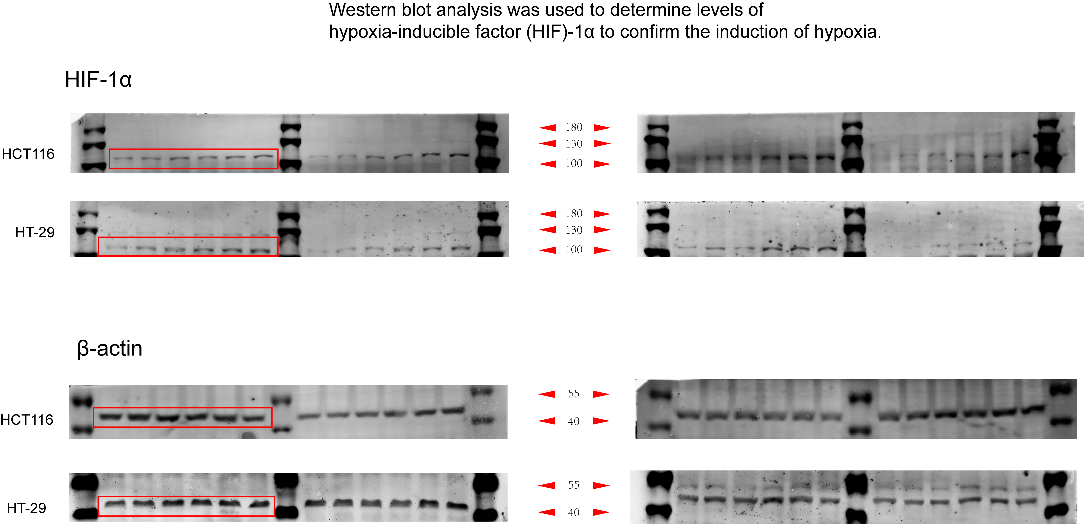


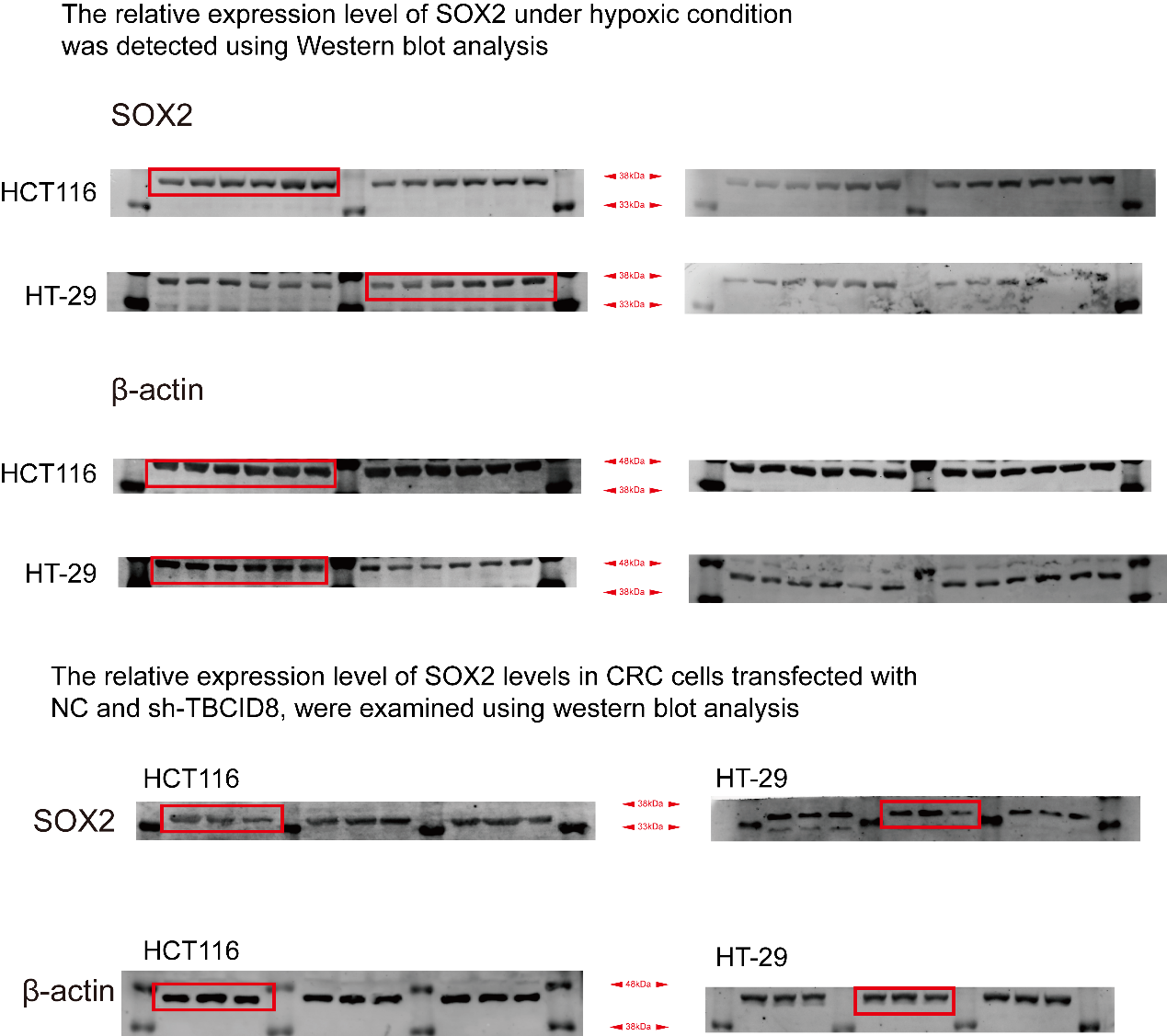

Supplement: Supplementary file 1 — Supplementary Information. [file 41598_2022_15629_MOESM1_ESM.docx]
